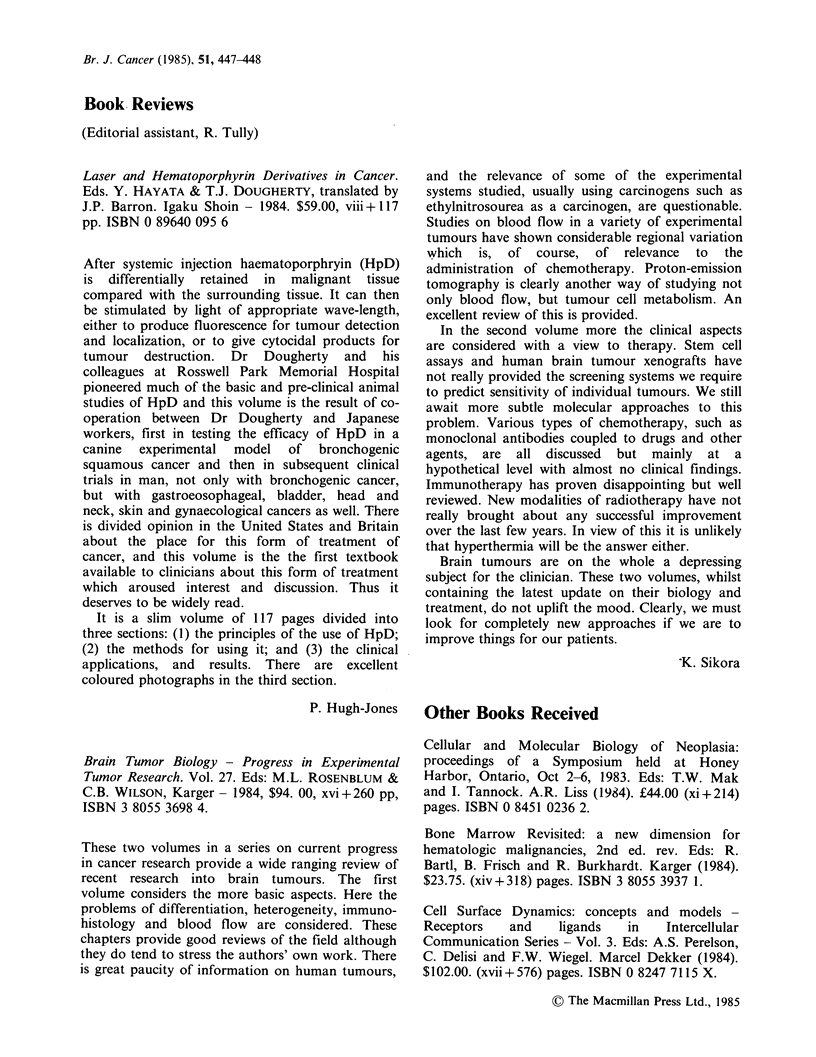# Laser and Hematoporphyrin Derivatives in Cancer

**Published:** 1985-03

**Authors:** P. Hugh-Jones


*Br. J. Cancer* (1985), **51**, 447–448

### Book Reviews

(Editorial assistant, R. Tully)

*Laser and Hematoporphyrin Derivatives in Cancer.* Eds. Y. HAYATA & T.J. DOUGHERTY, translated by J.P. Barron. Igaku Shoin – 1984. $59.00, viii + 117 pp. ISBN 0 89640 095 6

After systemic injection haematoporphryin (HpD) is differentially retained in malignant tissue compared with the surrounding tissue. It can then be stimulated by light of appropriate wave-length, either to produce fluorescence for tumour detection and localization, or to give cytocidal products for tumour destruction. Dr Dougherty and his colleagues at Rosswell Park Memorial Hospital pioneered much of the basic and pre-clinical animal studies of HpD and this volume is the result of co-operation between Dr Dougherty and Japanese workers, first in testing the efficacy of HpD in a canine experimental model of bronchogenic squamous cancer and then in subsequent clinical trials in man, not only with bronchogenic cancer, but with gastroeosophageal, bladder, head and neck, skin and gynaecological cancers as well. There is divided opinion in the United States and Britain about the place for this form of treatment of cancer, and this volume is the the first textbook available to clinicians about this form of treatment which aroused interest and discussion. Thus it deserves to be widely read.

It is a slim volume of 117 pages divided into three sections: (1) the principles of the use of HpD; (2) the methods for using it; and (3) the clinical applications, and results. There are excellent coloured photographs in the third section.

P. Hugh-Jones